# The efficacy and safety of Apatinib in the treatment of advanced non-small cell lung cancer: A retrospective trial

**DOI:** 10.3389/fonc.2022.1030798

**Published:** 2022-11-23

**Authors:** Jijin Wang, Di Huang, Wenjing Yang, Qingxu Song, Yibin Jia, Pengxiang Chen, Yufeng Cheng

**Affiliations:** Department of Radiation Oncology, Qilu Hospital of Shandong University, Jinan, Shandong, China

**Keywords:** advanced non-small cell lung cancer, anti-angiogenesis, apatinib, safety, survival

## Abstract

**Background:**

As a potent inhibitor of the vascular endothelial growth factor (VEGF) signaling pathway, Apatinib has been used in antitumor treatment for some time. The study aimed to research the therapeutic effects and toxicity of Apatinib in the treatment of advanced non-small cell lung cancer (NSCLC).

**Methods:**

We retrospectively analyzed 128 NSCLC patients treated with Apatinib in Qilu Hospital of Shandong University. Response Evaluation Criteria in Solid Tumors (RECIST) criteria was adopted to evaluate the treatment effect, and Common Terminology Criteria for Adverse Events (CTCAE) version 4.0 was conducted to determine the Adverse Events (AEs). Cox proportional hazard model and Kaplan-Meier function were applied to evaluate the progression-free survival (PFS) and overall survival (OS).

**Results:**

Among 128 NSCLC patients, partial response (PR) were observed in 15 patients, stable disease (SD) in 66 patients and progressive disease (PD) in 47 patients. The objective response rate (ORR) and disease control rate (DCR) accounted for 11.7% and 63.3% respectively. The median PFS (mPFS) and median OS (mOS) were 4.4 months and 17.2 months. Common side effects of Apatinib were hypertension (n=48), proteinuria (n=35), and hand-foot syndrome (HFS) (n=30), all of the side effects were controllable. No significant difference was observed in efficacy and AEs between the higher dose group (Apatinib>500mg/d) and the lower dose group (Apatinib=500mg/d).

**Conclusions:**

The study suggested that Apatinib with a lower dose (=500mg/d) has good efficacy and safety in the treatment of advanced NSCLC after first-line chemotherapy.

## Introduction

The morbidity and mortality rates of lung cancer have been staying at the top among all types of cancers for decades of years (1), and the incidence has been climbing year by year. There are two dominant subgroups of lung cancer, including small cell lung cancer (SCLC) and non-small cell lung cancer (NSCLC), and the latter accounts for 85% (2). The majority of advanced NSCLC patients have lost the opportunity of radical surgery or radiotherapy because of advanced stages at diagnosis, while chemotherapy, targeted therapy, and immunotherapy constitute the main treatment regimens for metastatic NSCLC (3). As known that almost all treatment regimens will develop resistance sooner or later. With the limited effect of traditional remedies, the 5-year survival rate for metastatic NSCLC is less than 10% (2). Therefore, there is an urgent need to explore new targeted therapies to improve the prognosis of advanced NSCLC.

Since Folkman proposed the theory of “Antiangiogenesis” in 1971 (4), several clinical researches had been conducted to probe into the detailed mechanisms of anti-angiogenesis and try to determine the efficacy and safety of targeted drugs. As reported, VEGF/VEGF receptor-2 (VEGFR-2) is the main signal pathway of VEGF-induced pathological angiogenesis (5). As a novel, small molecule, oral, antiangiogenic agent, Apatinib can not only inhibit the VEGF signal pathway by targeting the intracellular ATP binding site of tyrosine kinase, but also act on the signal bypass of tyrosine kinase inhibitors (TKIs), such as c-kit, ret, and src (6). Anti-angiogenesis therapy targeting VEGFRs has shown great efficacy in a variety of solid tumors, such as breast, renal, and hepatic cancers (7–9).

Based on two large clinical trials, China Food and Drug Administration (CFDA) has approved Apatinib, with a recommended dose of 750 mg/d, in the treatment of advanced gastric and gastroesophageal junction adenocarcinoma in 2014 (10, 11). Meanwhile, several researches have discussed the efficacy and safety of Apatinib in the treatment of NSCLC (12), but the appropriate therapeutic dose has not been confirmed yet. Our research intended to reiterate the efficacy and toxicity of Apatinib, and also to find a preferable dosage with satisfactory curative effect and tolerable side effects for advanced NSCLC.

## Materials and methods

### Patients

We retrospectively reviewed 128 patients diagnosed with advanced NSCLC in Qilu Hospital of Shandong University between June 1, 2016 and March 31, 2018. All the patients received Apatinib treatment after the failure of first or second-line chemotherapy (with or without EGFR targeted therapy) based on platinum. The inclusion criteria include (1): age between 18 and 85 when diagnosis (2); pathologically or cytologically confirmed advanced NSCLC (stage III or IV) without the chance of surgery (3); failure after at least the first line of systemic treatment or intolerable to standard chemotherapy (4); at least one measurable lesion defined by RECIST criteria (5); an Eastern Cooperative Oncology Group performance status (ECOG PS) of 0 to 2 (6); adequate hematologic, hepatic, and renal function. People with uncontrolled blood pressure or with bleeding tendency or those receiving thrombolytics or anticoagulants were excluded. Those who were pregnant, lactating, or had received other VEGFR-2 TKIs previously were also excluded from this study.

This study met the standards of the Declaration of Helsinki and was approved by the Ethics Committee of the Qilu Hospital.

### Treatment methods

Considering patients’ ECOG PS and physical differences, doctors determined the reasonable Apatinib mesylate (Jiangsu Hengrui Medicine, Jiangsu, China) dosage ranging from 250mg bid to 850mg qd. Patients who received Apatinib doses of 250mg bid and 500mg qd were grouped into the low-dose of Apatinib group, and who received Apatinib doses of 425mg bid, 625mg qd, and 850mg qd were grouped into the high-dose of Apatinib group. The treatment cycle was 28 days. Patients were expected to take Apatinib 30 minutes after the meal until disease progression, intolerable adverse events (AEs), withdrawal, or death. No radiotherapy, chemotherapy, or other local therapy was conducted during the period of Apatinib treatment, but supportive treatments were allowed for the management of AEs.

### Evaluation of efficacy and toxicity

Tumor responses were assessed by magnetic resonance imaging (MRI) or computed tomography (CT) after two cycles of treatment or significant signs of progression appeared or necessary. Blood pressure was checked daily during treatment, blood routine examination and biochemical parameters were tested weekly, and urine and stool were tested biweekly. According to RECIST version 1.1, tumor responses included complete response (CR), partial response (PR), stable disease (SD) and progressive disease (PD). The objective response rate (ORR) was defined as the percentage of patients who can be evaluated and reached for CR or PR at least 4 weeks. The disease control rate (DCR) was defined as the addition of objective response and stabilization rates (CR+PR+SD). Progression-free survival (PFS) was defined as the time period from initiating Apatinib treatment to tumor progression or death or loss of follow-up. Overall survival (OS) was defined as the time period from initiating Apatinib treatment to the date of death of any cause or last follow-up. AEs were reviewed from patients’ medical history and laboratory examinations and divided into grades 0-IV according to CTCAE version 4.0.

### Statistical analysis

Correlations between categorical variables and the short-term efficacy were assessed by the Chi-squared test or Fisher’s exact test, when appropriate. The Cox proportional hazards regression model (univariate and multivariate analyses) was adopted in the assessment of prognostic factors. Survival analysis was conducted by the Kaplan-Meier method with log-rank test. A two-sided *P*-value <0.05 was used in this study and considered statistically significant. All statistical analyses were performed using SPSS 23.0 statistical software (SPSS, Inc., Chicago, IL).

## Results

### Clinicopathological characteristics in patients with advanced NSCLC

A total of 128 patients diagnosed with NSCLC by pathology were included. The clinicopathological characteristics are shown in **Table 1**. The median age of patients was 60.5 years. The majority of patients were males (n=77, 60.2%) meanwhile the number of females was 51 accounting for 39.8%. In addition, 69 patients had ECOG PS of 0-1 score, 59 patients had ECOG PS of 2 scores. Sixty-three patients had never smoked, and 65 patients had a history of smoking. The EGFR status was examined in 35 of the 128 patients, with mutant type in 10 patients (7.8%), and wild-type in 25 patients (19.5%). The numbers of adenocarcinoma, squamous cell carcinoma, and the others (except SCLC) were 90, 16, and 22 respectively. Ten patients received oral Apatinib as second-line therapy and 118 as further-line treatment. Sixty-one patients were at the TNM stage III and 67 patients at stage IV. Twenty-four patients (18.7%) were grouped into the high-dose Apatinib group, while 104 patients (81.3%) were grouped into the low-dose Apatinib group.

**Table 1:** Patients clinical and pathological characteristics.

**Characteristics**	** **	**Patients (n [%])**
Gender	Female	51 (39.8)
	Male	77 (60.2)
		
Age (years)	<60	58 (45.3)
	≥60	70 (54.7)
ECOG PS	≤1	69 (53.9)
	2	59 (46.1)
Smoking history	No	63 (49.2)
	Yes	65 (50.8)
EGFR status	Wild-type	25 (19.5)
	Mutation	10 (7.8)
	Unknown	93 (72.7)
Number of metastases	0	33 (25.8)
	≤2	54 (42.2)
	≥3	41 (32.0)
Pre-radiotherapy	No	54 (42.2)
	Yes	74 (57.9)
Pathology	AD	90 (70.3)
	SCC	16 (12.5)
	Others	22 (17.2)
Apatinib dose (mg/d)	-500	104 (81.3)
	>500	24 (18.7)
Line of Apatinib	Second line	10 (7.8)
	Further line	118 (92.2)
TNM stage	III	61 (47.7)
	IV	67 (52.3)
Hypertension during treatment	No	80 (62.5)
	Yes	48 (38.4)
Proteinuria during treatment	No	93 (72.7)
	Yes	35 (27.3)
HFS during treatment	No	98 (76.6)
	Yes	30 (23.4)

1 ECOG PS, Eastern Cooperative Oncology Group performance status; 2 AD, adenocarcinoma;3 SCC, squamous cell carcinoma; 4 TNM, tumor, node, and metastases; 5 HFS, hand-foot syndrome.

### Treatment efficacy

**Figure 1:**
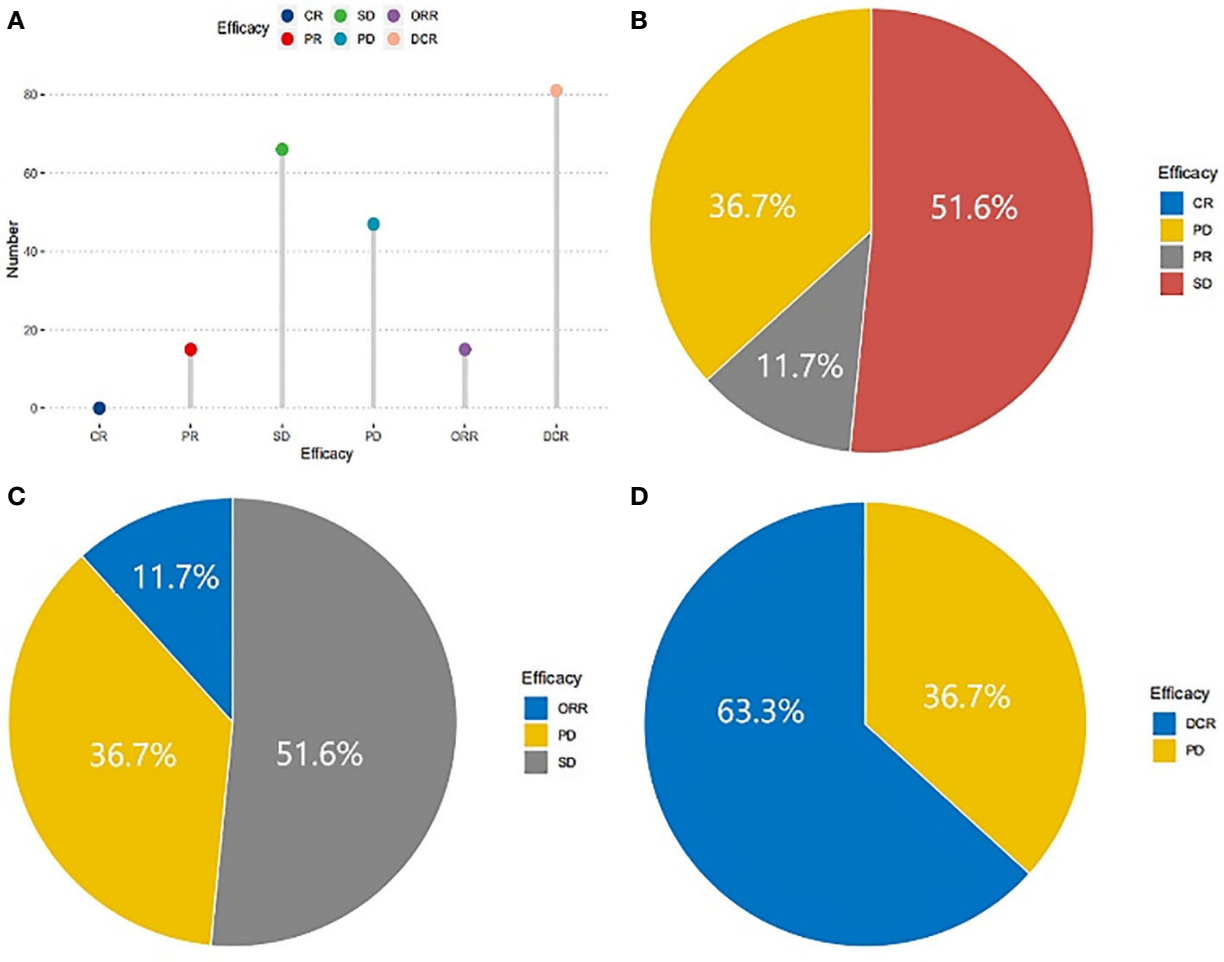
Short-term efficacy of patients in NSCLC treated with Apatinib. No patient with CR, 15 patients with PR, 66 patients with SD and 47 with PD **(A)**, the proportions were 0, 36.7%, 11.7%, 51.6% respectively **(B)**. The ORR was 11.7% **(C)** and DCR was 63.3% **(D)**.

Followed-up was completed on February 1, 2019, and the short-term efficacy was shown in **Figure 1**. All patients met the criteria for response assessment. No patient achieved CR, while15, 66, and 47 patients reached PR, SD, and PD, respectively (**Figures 1A, B**). The ORR was 11.7% (**Figure 1C**) and DCR was 63.3% (**Figure 1D**). The correlations between clinicopathological variables and short-term efficacy were explored by logistic regression analysis. As shown in **Supplementary Table 1**, smoking history, TNM stage (III/IV), and hypertension in the treatment were related to ORR, with *P*-values of 0.025, 0.014, and 0.005, respectively. ECOG PS (*P <*0.001), smoking history (*P <*0.001), No. of metastasis (*P* =0.002), TNM stage (*P <*0.001), and hypertension (*P <*0.001) in the treatment showed correlations with DCR. Then the significant variables identified by univariate analysis were put into multivariate analysis (**Supplementary Table 1**), and the results exhibited that patients with hypertension (OR 5.649, *P* =0.005) were more likely to achieve ORR, and ECOG PS (OR 0.171, *P* =0.013), smoking history (OR 0.124, *P* =0.006), and TNM stage (OR 0.228, *P* =0.022) were risk factors for DCR. Different dose of Apatinib showed no impact for both ORR and DCR.

**Figure 2:**
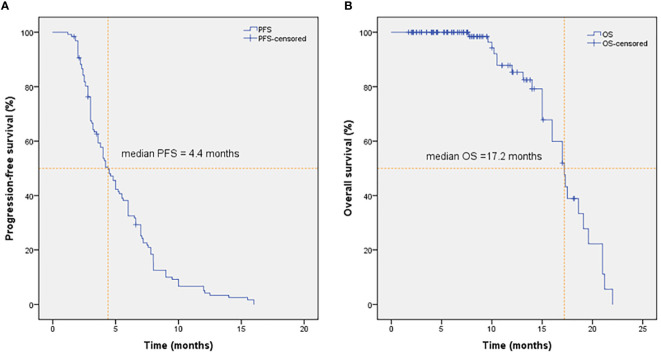
PFS **(A)** and OS **(B)** of patients in NSCLC treated with Apatinib. The mPFS and mOS were 4.4 months and 17.2 months in NSCLC treated with Apatinib, respectively.

**Figure 3:**
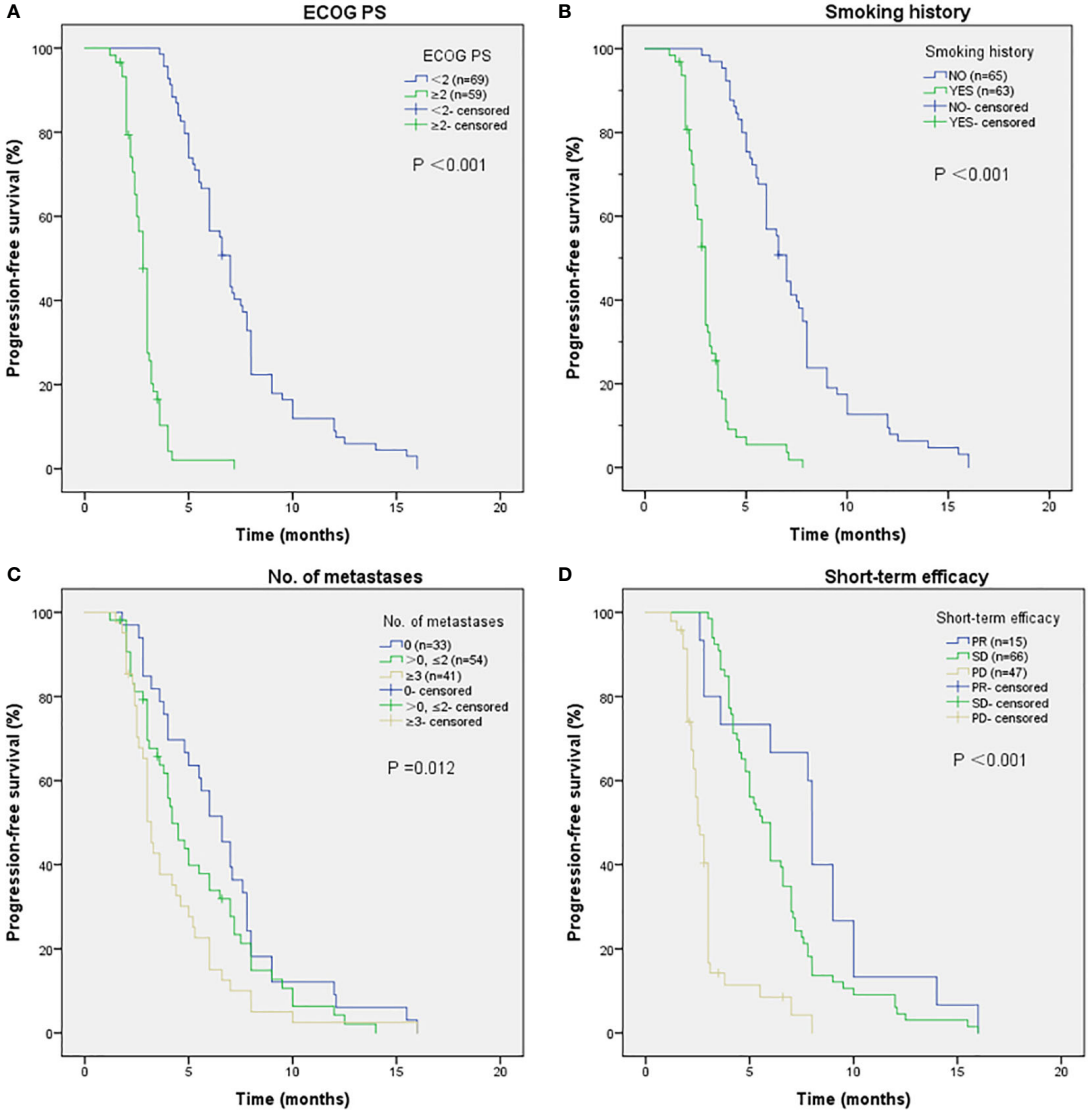
The relationship between ECOG PS and PFS **(A)**, with *P-*value <0.001. Patients with smoking history have poorer PFS **(B)**, with *P-*value <0.001. The relationship between No. of metastases and PFS **(C)**, with *P-*value of 0.012. The relationship between short-term efficacy and PFS **(D)**. No people reached CR, people who reached PR had better PFS results than people who suffered SD and PD, with *P-*value <0.001.

**Figure 4:**
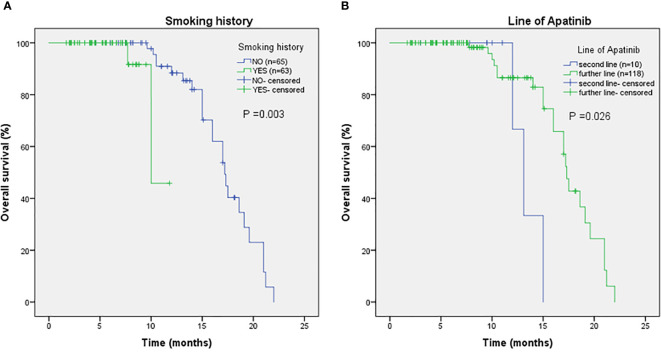
The relationship between smoking history and OS **(A)**, with *P-*value of 0.003. The relationship between line of Apatinib and OS **(B)**, with *P-*value of 0.026.

As shown in **Figure 2**, the mPFS of patients was 4.4 months (range from 1.2-16.0 months) and the mOS was 17.2 months (range from 1.7-22.0 months). Univariate (**Table 2**) and multivariate cox regression analysis (**Table 3**) were used to evaluate the relationship between survival outcomes and patients’ characteristics in advanced NSCLC. The results showed that ECOG PS (HR 6.947, *P <*0.001), smoking history (HR 4.076, *P <*0.001), No. of metastasis (HR 1.356, *P* =0.024), and short-term efficacy (HR 2.188, *P <*0.001) were independent prognostic factors for PFS. Meanwhile, smoking history (HR 14.003, *P* =0.007) and line of Apatinib (HR 0.219, *P* =0.028) were independent prognostic factors for OS. There was no statistical difference in different dose groups of Apatinib for PFS. It’s important to emphasize that Apatinib dose was associated with OS in the univariate analysis (HR 2.923, *P* =0.049), but not as an independent prognostic factor in the multivariate analysis. **Figure 3** detailed the variables that have an impact on PFS by the Kaplan-Meier method. And **Figure 4** exhibited the relationship between significant variables and OS, which showed that patients with smoking history had poorer OS (*P* =0.003) than patients who never smoked, and Apatinib used as a second-line drug had worse OS than as a further-line drug, with a *P*-value of 0.026.

**Table 2:** Univariate analysis of PFS and OS.

Characteristics	PFS		OS	
	HR (95% CI)	*P*-value	HR (95% CI)	*P*-value
Gender (Female/Male)	0.943 (0.655-1.357)	0.753	0.694 (0.303-1.590)	0.388
Age (<60/≥60, years)	0.960 (0.673-1.370)	0.822	0.827 (0.365-1.873)	0.650
ECOG PS (<2/≥2)	13.103 (7.924-21.667)	<0.001*	3.334 (0.375-29.662)	0.280
Smoking history	7.468 (4.782-11.662)	<0.001*	11.021 (1.754-69.242)	0.010*
EGFR status (Wild/Mutation/Unknown)	1.054 (0.840-1.321)	0.651	0.870 (0.546-1.387)	0.558
No. of metastases (0/≤2/≥3)	1.395 (1.103-1.764)	0.005*	1.125 (0.669-1.891)	0.657
Pre-radiotherapy	1.039 (0.726-1.488)	0.834	0.741 (0.336-1.634)	0.457
Pathology (AD/SCC/Others)	1.049 (0.827-1.332)	0.692	0.664 (0.314-1.404)	0.284
Apatinib dose (=500/>500, mg/d)	1.309 (0.828-2.069)	0.250	2.923 (1.006-8.492)	0.049*
Line of Apatinib (Second line/Further line)	0.969 (0.504-1.864)	0.924	0.252 (0.067-0.949)	0.042*
TNM stage (III/IV)	2.858 (1.939-4.211)	<0.001*	1.427 (0.466-4.369)	0.534
Short-term efficacy (PR/SD/PD)	3.223 (2.277-4.562)	<0.001*	1.162 (0.454-2.972)	0.754
Hypertension	0.290 (0.190-0.442)	<0.001*	0.635 (0.175-2.310)	0.491
Proteinuria	1.230 (0.815-1.855)	0.325	1.310 (0.475-3.614)	0.602
HFS	0.933 (0.617-1.411)	0.743	1.580 (0.669-3.730)	0.297

PFS, progression-free survival; OS, overall survival; HR, hazard ratio; CI, confidence interval; ECOG PS, Eastern Cooperative Oncology Group performance status; AD, adenocarcinoma; SCC, squamous cell carcinoma; TNM, tumor, node, and metastases; PR, partial response; SD, stable disease; PD, progressive disease; HFS, hand-foot syndrome.

*Statistically significant values, P <0.05.

**Table 3:** Multivariate analysis of PFS and OS in patients with NSCLC.

Characteristics	PFS		OS	
	HR (95%CI)	*P*-value	HR (95%CI)	*P*-value
ECOG PS (<2/≥2)	6.947 (3.657-13.194)	<0.001*	–	–
Smoking history	4.076 (2.238-7.427)	<0.001*	14.003 (2.038-96.204)	0.007*
No. of metastases (0/≤2/≥3)	1.356 (1.040-1.769)	0.024*	–	–
Line of Apatinib (Second line/Further line)	–	–	0.219 (0.057-0.846)	0.028*
Short-term efficacy (PR/SD/PD)	2.188 (1.483-3.226)	<0.001*	–	–

PFS, progression-free survival; OS, overall survival; HR, hazard ratio; CI, confidence interval; ECOG PS, Eastern Cooperative Oncology Group performance status; PR, partial response; SD, stable disease; PD, progressive disease.

*Statistically significant values, P <0.05.

### Safety assessment

The incidences of different grades of AEs were presented in **Table 4**. All adverse reactions were restricted to grade 1 to grade 3, no grade 4 treatment-related AEs were observed in the present study. Hypertension, proteinuria, and hand-foot syndrome (HFS) were the first three common side effects and accounting for 37.5%, 27.3%, and 23.4%, respectively. Fatigue (15.2%), hematologic toxicities (16.4%), anorexia (15.6%), and nausea/vomiting (14.8%) were even more rare. Furthermore, drug-related total AEs and grade 3 AEs in different dose groups of Apatinib were listed and analyzed by Pearson’s chi-square method. The results showed that there was no significant difference in AEs between different Apatinib doses, with the *P*-values of <0.05. All of the patients could tolerate the side effects with supportive care, and no deaths associated with Apatinib treatment were observed.

**Table 4:** Treatment-related AEs in patients with advanced NSCLC.

AEs	Total patients (n=128)		Apatinib dose = 500mg/d (n=104)		Apatinib dose > 500mg/d (n=24)	
	Total	Grade3	Total	Grade3	Total	Grade3
Hypertension	48 (37.5%)	7 (5.5%)	39 (37.5%)	4 (3.8%)	9 (37.5%)	3 (12.5%)
Proteinuria	35 (27.3%)	6 (4.7%)	31 (29.8%)	4 (3.8%)	4 (16.7%)	2 (8.3%)
HFS	30 (23.4%)	0 (0.0%)	24 (23.1%)	0 (0.0%)	6 (25.0%)	0 (0.0%)
Fatigue	24 (15.2%)	2 (1.6%)	18 (17.3%)	2 (1.9%)	6 (25.0%)	0 (0.0%)
Hematologic toxicities	21 (16.4%)	5 (3.9%)	16 (15.4%)	4 (3.8%)	5 (20.8%)	1 (4.2%)
Anorexia	21 (15.6%)	3 (2.3%)	15 (14.4%)	2 (1.9%)	6 (25.0%)	1 (4.2%)
Nausea and vomiting	19 (14.8%)	1 (0.8%)	13 (12.5%)	0 (0.0%)	6 (25.0%)	1(4.2%)

AEs, adverse effects; HFS, hand-foot syndrome.

## Discussion

Apatinib has a wide effective dose scope of 250mg-850mg and targeted the VEGF/VEFGR2 pathway (5, 13). Previous studies have reported that Apatinib is an effective treatment in the development of several solid tumors including advanced NSCLC (8, 12). The present study reported the efficacy and safety of Apatinib as second- or further-line therapy in advanced NSCLC patients. The results showed that the ORR and DCR were11.7% and 63.3%, and the median PFS and OS were 4.4 and 17.2months, respectively. There was no grade 4 toxicity, and all of the side effects could be manageable and tolerable. Additionally, we found that low dose of Apatinib seem to provide further benefits for OS, without considering the influence of other factors. The AEs in the low-dose Apatinib group was similar to that of the high-dose group.

The ORR (11.7%) and DCR (63.3%) in our study were better than the standard therapeutic regimens based on docetaxel, with the ORR and DCR were 8.8% and 55.2%, respectively (14). It has been reported that patients with positive EGFR mutations can achieve impressive efficacy with an ORR of 51.5% and DCR of 91% when combined with Apatinib after resistance to EGFR-TKI (15). Since EGFR and VEGFR have parallel and common downstream signaling pathways, especially during the angiogenesis phase, Apatinib can reverse multidrug resistance (MDR) including EGFR-TKI (16, 17). This means Apatinib combined with EGFR-TKIs could produce a better result after the resistance to EGFR-TKIs. In our study, the proportion of patients with EGFR mutations was 7.8%. This group of patients used EGFR-TKI in first-line or second-line treatment but changed to Apatinib monotherapy after drug resistance. Therefore, in the subgroup analysis, no better benefit was seen in patients with EGFR mutations.

A phase II clinical trial from Wu (18), in which patients received 500 or 750 mg Apatinib daily, reported that Apatinib provided a promising efficacy with median PFS of 3.06 months and OS of 7.69 months in advanced NSCLC. The side effects were also manageable, but the authors did not explore the effect of different doses of Apatinib on survival. In recent years, there have been many studies on Apatinib combined with cytotoxic drugs, radiotherapy or immunotherapy (19–22). Zhou (23) investigated the efficacy of Apatinib in combination with Camrelizumab in patients with advanced non-squamous NSCLC. The results showed that 30.9% of patients reached ORR, the mPFS was 5.7 months and mOS was 15.5 months. The efficacy of the study above is far better than ours, as the mPFS and mOS in our study were 4.4 months and 17.2 months. Due to the retrospective nature of our study, we were unable to evaluate the synergistic effect of Apatinib with immunological agents in this patient cohort. However, the population of NSCLC patients is huge, and there are still many patients who are not suitable or unable to use immune agents. Our study has guiding significance in this group of patients. A study of Apatinib in patients with breast cancer reflected that both high expression of phosphorylated VEGFR2 and hypertension were potential predictive indicators for treatment efficacy (24). Univariate analysis of our study also found the predictive effect of hypertension (HR=0.290, *P <*0.001) for PFS. Our study, limited by the retrospective nature, had no status of VEGFR2 detected, thus there was no exploration on the correlation between VEGFR2 and survival.

Low dose and high dose of Aptinib may have different mechanisms, and it has been reported that low dose of Apatinib can improve the immune microenvironment of lung cancer (25). However, the appropriate dose of Aptinib has not been explored in case-control studies. Our study examined the effect of different doses of Apatinib on short-term efficacy and survival, the results showed that low dose of Apatinib may favor a better OS, while the effect of different doses of Apatinib on short-term efficacy did not differ. We expected that the increased side effects of the high-dose drug would offset some of the survival benefit of the patients, but subsequent analysis found that there was no significant difference in toxicity between the two groups. Therefore, the OS benefit of low-dose drugs was not from the decreasing toxicity and the mechanism behind needs further research, including basic mechanism research and clinical trials with expanded sample size.

This research not only evaluated the efficacy and safety of Apatinib in advanced NSCLC but also tried to determine a reasonable dose of Apatinib to make it more valuable with fewer side effects. The top three AEs observed in our study included hypertension, proteinuria, and HFS, but only seven patients (5.5%) showed grade 3 hypertension and six patients (4.7%) showed grade 3 proteinuria. The lower incidence of grade 3 AEs associated with antiangiogenesis may be due to the higher selectivity of Apatinib for VEGFR2 compared with other antiangiogenic drugs (26). The incidence of total toxicity and grade 3 toxicity were similar between the low-dose Apatinib group and high-dose Apatinib group. All of the side effects can be relieved by symptomatic treatment and support care, suggesting that Apatinib monotherapy was of great safety in the treatment of advanced NSCLC. Considering the economic principle and patient compliance, our study showed that a lower dose of Apatinib (500mg/d) might be a feasible strategy in the treatment of advanced NSCLC after at least one cycle of treatment. Reducing the financial burden on patients may improve their quality of life, and enhancing their oral taking compliance may translate into survival benefits in the long run.

However, there were too few patients in the high-dose Apatinib group than low-dose group, further large-scale clinical trials and prospective studies are needed to verify the conclusion. Meanwhile, how the lower-dose group translate into survival benefits needs to be further verified.

## Conclusions

In summary, our research demonstrated the high efficacy and safety of Apatinib in patients with advanced NSCLC, especially when they suffered progression after first-line chemotherapy. And also emphasized the superiority of lower dose Apatinib in the treatment strategy.

## Data availability statement

The raw data supporting the conclusions of this article will be made available by the authors, without undue reservation.

## Ethics statement

Written informed consent was obtained from the individual(s) for the publication of any potentially identifiable images or data included in this article.

## Author contributions

YC directed the project, and revised the paper. JW conceptualized and designed the study, analyzed the data, and wrote the paper. DH designed the study and analyzed the data. WY contributed useful suggestions and collected follow-up information. QS contributed useful suggestions. YJ analyzed the data. PC optimized the language. All authors read and approved the final manuscript.

## Funding

This study was supported by the National Natural Science Foundation of China (82172664 and 81972850) and Special Fund for Taishan Scholar Project (ts20190973). Natural Science Foundation of Shandong Province Biomedicine Joint Fund (ZR2021LSW020).

## Conflict of interest

The authors declare that the research was conducted in the absence of any commercial or financial relationships that could be construed as a potential conflict of interest.

## Publisher’s note

All claims expressed in this article are solely those of the authors and do not necessarily represent those of their affiliated organizations, or those of the publisher, the editors and the reviewers. Any product that may be evaluated in this article, or claim that may be made by its manufacturer, is not guaranteed or endorsed by the publisher.
