# Supplementary material for: The efficacy and safety of Apatinib in the treatment of advanced non-small cell lung cancer: A retrospective trial
Source: Front Oncol. 2022 Nov 23;12:1030798. doi: 10.3389/fonc.2022.1030798 (PMC9727187; doi:10.3389/fonc.2022.1030798)
Supplement: Supplementary file 1 [file DataSheet_1.docx]

**Supplementary Table 1 Univariate** **Logistic Regression Analysis of short-term efficacy**

| **Characteristics** | **ORR** | | **DCR** | |
| --- | --- | --- | --- | --- |
|  | **OR (95% CI)** | ***P*-value** | **OR (95% CI)** | ***P*-value** |
| Gender (Female/ Male) | 1.958 (0.588-6.528) | 0.274 | 0.903 (0.432-1.885) | 0.786 |
| Age (<60/ ≥60, years) | 1.279 (0.427-3.831) | 0.661 | 1.100 (0.535-2.263) | 0.796 |
| ECOG PS (<2/ ≥2) | 0.383 (0.115-1.276) | 0.118 | 0.032 (0.011-0.092) | <0.001* |
| Smoking history | 0.221 (0.159-0.825) | 0.025* | 0.030 (0.010-0.096) | <0.001* |
| EGFR status (Wild/ Mutation/ Unknow) | 0.896 (0.469-1.713) | 0.740 | 1.502 (0.966-2.335) | 0.071 |
| No. of metastases (0/ ≤2/ ≥3) | 0.775 (0.380-1.581) | 0.484 | 0.443 (0.264-0.744) | 0.002* |
| Pre-radiotherapy | 0.814 (0.276-2.399) | 0.709 | 0.674 (0.322-1.410) | 0.295 |
| Pathology (AD/ SCC/ Others) | 0.996 (0.494-2.007) | 0.709 | 0.847 (0.535-1.342) | 0.480 |
| Apatinib dose (=500/ >500, mg/d) | 0.636 (0.134-3.028) | 0.570 | 0.626 (0.255-1.537) | 0.307 |
| Line of Apatinib (Second line/ Further line) | 1.212 (0.143-10.297) | 0.860 | 0.174 (0.021-1.419) | 0.102 |
| TNM stage (III/ IV) | 0.191 (0.051-0.716) | 0.014* | 0.159 (0.069-0.365) | <0.001* |
| Hypertension | 5.649 (1.685-18.942) | 0.005* | 12.784 (4.197-38.943) | <0.001* |
| Proteinuria | 0.962 (0.285-3.247) | 0.950 | 0.825 (0.371-1.833) | 0.637 |
| HFS | 1.760 (0.551-5.624) | 0.340 | 1.478 (0.613-3.564) | 0.385 |

ORR, objective response rate; DCR, disease control rate; OR, odds ratio; CI, confidence interval; ECOG PS, Eastern Cooperative Oncology Group performance status; AD, adenocarcinoma; SCC, squamous cell carcinoma; TNM, tumor, node, and metastases; HFS, hand-foot syndrome.

*Statistically significant values, *P* <0.05.

**Supplementary Table 2 Multivariate Logistic Regression Analysis of short-term efficacy**

| **Characteristics** | **ORR** | | **DCR** | |
| --- | --- | --- | --- | --- |
|  | **OR (95%CI)** | ***P*-value** | **OR (95%CI)** | ***P*-value** |
| ECOG PS (<2/ ≥2) | - | - | 0.171 (0.042-0.690) | 0.013* |
| Smoking history | - | - | 0.124 (0.028-0.542) | 0.006* |
| TNM stage | - | - | 0.288 (0.099-0.839) | 0.022* |
| Hypertension | 5.649 (1.685-18.942) | 0.005* | - | - |

ORR, objective response rate; DCR, disease control rate; OR, odds ratio; CI, confidence interval; ECOG PS, Eastern Cooperative Oncology Group performance status; TNM, tumor, node, and metastases.

*Statistically significant values, *P* <0.05.
